# Indoor Visible Light Positioning: Overcoming the Practical Limitations of the Quadrant Angular Diversity Aperture Receiver (QADA) by Using the Two-Stage QADA-Plus Receiver †

**DOI:** 10.3390/s19040956

**Published:** 2019-02-24

**Authors:** Stefanie Cincotta, Cuiwei He, Adrian Neild, Jean Armstrong

**Affiliations:** 1 Department of Electrical and Computer Systems Engineering, Faculty of Engineering, Monash University, Wellington Rd, Clayton 3800, Victoria, Australia; cuiwei.he@monash.edu (C.H.); jean.armstrong@monash.edu (J.A.); 2 Department of Mechanical and Aerospace Engineering, Faculty of Engineering, Monash University, Wellington Rd, Clayton 3800, Victoria, Australia; adrian.neild@monash.edu

**Keywords:** visible light positioning, indoor positioning, angle of arrival, QADA, QADA-plus, quadrant photodiode

## Abstract

Visible light positioning (VLP), using LED luminaires as beacons, is a promising solution to the growing demand for accurate indoor positioning. In this paper, we introduce a two-stage receiver that has been specifically designed for VLP. This receiver exploits the advantages of two different VLP receiver types: photodiodes and imaging sensors. In this new receiver design a quadrant angular diversity aperture (QADA) receiver is combined with an off-the-shelf camera to form a robust new receiver called QADA-plus. Results are presented for QADA that show the impact of noise and luminaire geometry on angle of arrival estimation accuracy and positioning accuracy. Detailed discussions highlight other potential sources of error for the QADA receiver and explain how the two-stage QADA-plus can overcome these issues.

## 1. Introduction

Indoor positioning is an important new technology, with a wide variety of applications such as pedestrian navigation, asset tracking, and autonomous robot movement. There has been a lot research dedicated to finding an accurate and economical positioning system, with many potential approaches being explored [1,2]. One approach with huge potential is to use visible light positioning (VLP) [3,4,5]. In VLP, white LEDS are used for both indoor illumination and high-speed data transmission. VLP can take advantage of this technology by utilizing the LED lighting as positioning beacons that transmit their location using visible light communication (VLC). LEDs are fast becoming the standard choice for indoor lighting due to their energy efficiency and long lifespan, thus they could potentially be found in all buildings in the future. This makes VLP an attractive technology due its low infrastructure cost and its potential to achieve high levels of positioning accuracy.

The recent interest in VLP has resulted in an explosion in the number of papers describing a wide range of different novel indoor positioning systems [4,6,7]. Despite this, so far, no VLP system has emerged that is as useful and versatile as the Global Positioning System (GPS). To understand the challenges that VLP must meet, it is useful to consider the characteristics of GPS.

A GPS receiver works anywhere in the world as long as it can receive signals from enough satellite-mounted GPS transmitters [8]. To operate, a GPS receiver does not require any other source of information, such as access to the internet, or prior knowledge about its current location. Each GPS satellite transmits data about its position and also accurate time information based on the atomic clock carried on each satellite. Each GPS receiver must decode the transmitted information so that it knows the position of each satellite when the signal was transmitted. It must also measure the time difference of arrival of signals from each transmitter. By combining this with information about the satellite position and time, the receiver can estimate its distance from each transmitter and using trilateration attain its current position.

The fundamental challenge for VLP is to design a system, which like GPS, can combine the functions of data receiver and position estimation and so provide accurate position information without access to external databases or stored information. As we will show in the following literature survey, camera based receivers can provide accurate angle-of-arrival (AOA) information, which can be used for triangulation, but the rate at which they can receive data is very limited so they must rely on additional information, while photodiode (PD) based receivers can receive very high speed data, but cannot provide very accurate estimates of distance or angle-of-arrival. The aim of the QADA-plus is to combine the benefits of each receiver type.

A number of camera-based positioning systems have been described [9,10]. To receive data, most camera-based systems depend on the properties of the rolling shutters which are found in the cameras of typical mobile phones. One paper of interest is [11], which provides careful experimental results, and also discusses in detail many practical aspects of this type of system. They identify as a key limitation the very low data rate that can be supported by the rolling shutter approach. They overcome this by assuming that the receiver has access to a database containing the position of each transmitter, and so each luminaire need only transmit an identifying code. Because of the limited data rate of the rolling shutter approach, relatively few different codes can be used, and the system is not scalable. The paper however demonstrates that image-based systems can provide accurate positioning, with an experimentally average error of 7 cm within a 1 m × 1 m area illuminated by five beacon LEDs. Other papers have also demonstrated that camera-based systems can achieve accurate positioning, with an average error of 3.2 cm [10]. A number of papers have noted that the accuracy depends strongly on the number of beacons that are visible in the image [10,11].

A large number of systems have been described using PD receivers. Many of these are based on measurements of received signal strength (RSS). A disadvantage of RSS systems is that they usually require precise knowledge of the transmit power and the radiation pattern of each luminaire. In practice this information may not be known and/or may change with time as the efficiency of the LEDs in the luminaire reduce with time. The RSS measured at the receiver has also been shown to be sensitive to light reflected from walls [12] and to the precise waveform of the transmitted signal [13]. One way to mitigate the effect of unknown RSS is to use fingerprinting to measure the RSS at positions within the environment and use this stored information in the position calculations [14].

The use of AOA overcomes some of the limitations of RSS. In particular most AOA systems do not require knowledge of the transmitted optical power or the radiation pattern of each luminaire. However, AOA receivers do have to be able to determine the direction of arrival of the light. One way of achieving this is to have PDs with different orientations. Cubic and other structures have been described in the literature [15,16,17]. Another approach is to use an aperture receiver in which directionality is achieved by having different offsets between the aperture and the PD [18]. It has been shown theoretically [19] that, if it is assumed that there is perfect orientation and positioning of PDs and perfect matching of the receiver electronics, centimeter accuracy can be achieved by these receivers. However, these are unrealistic constraints for a practical system. The QADA structure reduces these constraints by using one aperture and using a quadrant PD with closely matched quadrants, but it does not completely solve the problem, and there is still significant discrepancy between the theoretical results and experimental measurements even under laboratory conditions and short transmission distances [20]. In fact, this is an example of a fundamental limitation of systems using a small number of PDs: the accuracy is strongly dependent on the precise value of the measured signal for each PD.

Time-based systems [21], like the time difference of arrival (TDOA) method used in GPS, are not useful in VLP as they require extremely accurate synchronization. Another approach is to merge information from different sensors, for example information from both a camera and accelerometer is used in [22].

Camera-based positioning is also used in other fields, particularly surveying and robot systems. Particularly relevant is the work on single camera systems using fiducial markers [23,24]. These visual markers are similar to QR codes, but they are specially designed for positioning. Because they may form a relatively small portion of an image and yet must still be both recognizable and decodable, they can only carry a limited number of bits of information. For example, the Apriltag version with 10 checkbits can only have 2221 distinct markers [24], so, like camera-based VLP systems, access to a lookup table is required.

In summary, although many different indoor positioning systems have been proposed, none so far matches the functionality of the GPS system.

In [25] we introduced the quadrant angular diversity aperture (QADA) receiver. This receiver was designed to have a compact, planar structure with low power requirements so that it could be easily incorporated into devices like mobile phones. We have demonstrated that QADA is capable of estimating the AOA of the incoming light [20], however we have also shown that it is extremely sensitive to fabrication imperfection and it is unlikely to achieve the accuracy that is implied by simulation results.

**Figure 1:**
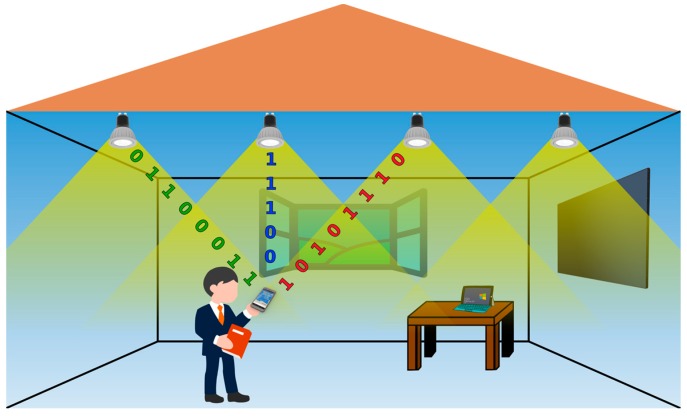
Typical indoor positioning scenario.

To overcome the challenges faced by QADA, we have developed QADA-plus [26]. This two-stage system provides an elegant solution by combining the QADA with a camera. Taking advantage of the strengths of both sensors, QADA-plus is able to perform well in a wide variety of indoor environments, making it an ideal VLP receiver. In Figure 1, a typical indoor scenario is shown where a person is receiving positioning information on a smart phone that has a QADA-plus. The LED luminaires serve two functions–room illumination and positioning beacons. Each luminaire is transmitting its coordinates, which is then decoded by the QADA and used, in combination with the camera data, to determine the position of the person holding the receiver. This is just one potential implementation of this new receiver design.

In this paper, we demonstrate the limitations of the QADA receiver by individually exploring several sources of error. At each stage we highlight the assumptions made in the simulation models and the other potential sources of error that will lead to further performance degradation.

The contributions in this paper are:

1. Detailed discussion of the potential sources of error for the QADA and how adopting the two-stage system will lead to better performance.
2. Description of reference points and their importance in precise indoor positioning.
3. In depth analysis of the impact of luminaire size and shape on the QADA receiver.
4. Triangulation results for the QADA that show how noise, luminaire shape and size and room dimensions impact on positioning accuracy.

## 2. Indoor Positioning with QADA and QADA-Plus

QADA-plus is a new form of two-stage receiver for VLP. A unique feature of this receiver is that it exploits the different advantages of PD-based receivers and camera-based receivers. This creates a hybrid receiver that is purpose designed for VLP. It consists of a QADA, which is ideal for receiving high-speed data with approximate AOA information, and a camera which captures high resolution images that are used to add accuracy to the estimated angles. Reference points are a further optional enhancement for use with QADA-plus that can improve accuracy.

### 2.1. QADA Design

**Figure 2:**
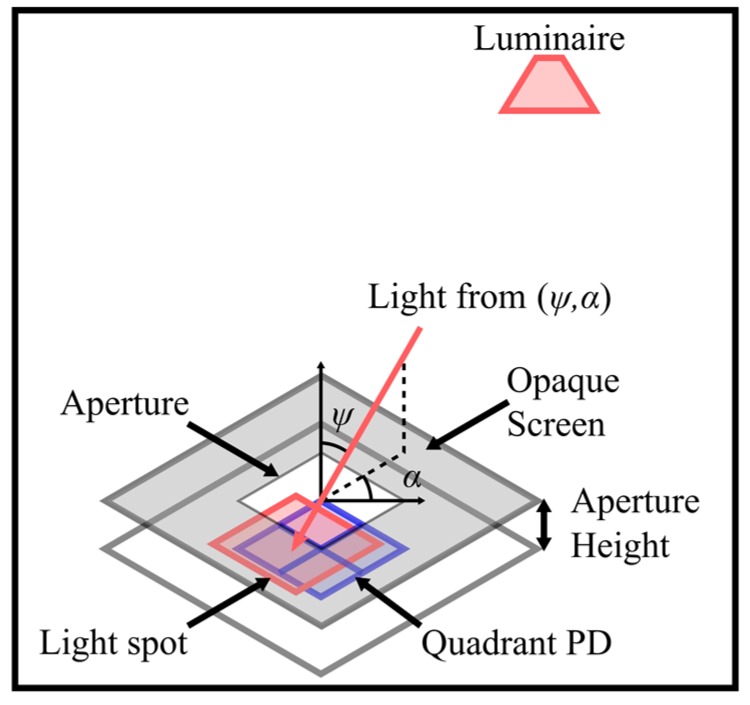
QADA receiver design.

This section details the design of QADA which is the first stage sensor in the QADA-plus receiver. QADA, shown in Figure 2, couples a quadrant PD with an aperture to create an angular diversity receiver that is compact and can be constructed from standard off-the-shelf components. A quadrant PD is made up of four individual PDs closely arranged in a 2 × 2 grid, with a small gap separating the quadrants. Unlike conventional applications, such as atomic force microscopy [27], that use quadrant PDs with laser light [28], our innovative use of an aperture means that QADA can be used with standard indoor illumination.

The location of the centroid of the light spot that falls on the quadrant PD unambiguously determines the AOA. An important feature is the known aperture height, which is the vertical distance separating the aperture plane from the quadrant PD plane. This aperture height is selected to determine the field of view (FOV) of the QADA and, because it is a known value, it can be combined with the centroid of the light spot to estimate the incident and polar angles of the light.

An important advantage of using a square shape for the quadrant PD and the aperture is that the area of the light spot which overlaps the PD changes linearly as it passes over the PD. The aperture size matches the quadrant PD size exactly to maximize the received light. It is not possible to use an aperture that is larger than the quadrant PD as this would lead to ambiguity when estimating the AOA.

### 2.2. QADA-Plus

For many applications that require only approximate localization QADA alone may be adequate. Although theoretical results for QADA in ideal conditions [20,25,26] show promising results, in practice, factors such as minor changes to the aperture or partial blockages of the light path to the luminaire limit the accuracy that can be achieved. In [20], we demonstrated with a carefully controlled small-scale experiment that there was a large discrepancy between experimental and simulation results. This highlighted the limitations of the simulation results and also of the ability of the QADA to provide precise positioning. For applications that do require high accuracy, QADA can be improved by adding a second stage imaging sensor to become QADA-plus. QADA is a high speed but low pixel sensor, that can offer moderate accuracy, whilst a camera is a low speed, but high pixel sensor that offers high accuracy. For the two-stage system, the requirements for a highly calibrated and accurate QADA are much lower. An important requirement is that the QADA is sufficiently accurate to be able to identify the luminaires. The precise image co-ordinates of the identified LED beacons are used in a triangulation algorithm like the one we detailed in [26].

**Figure 3:**
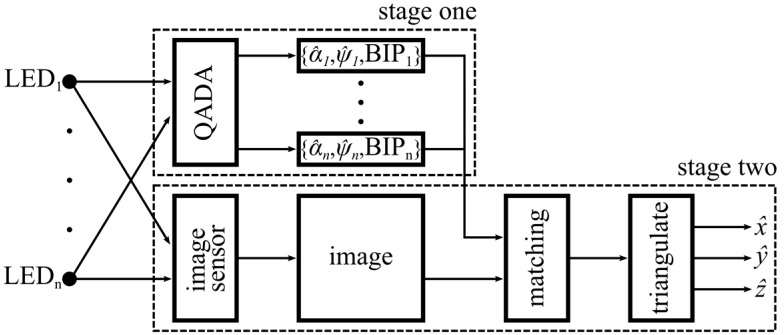
Block diagram showing the flow of information of QADA-plus.

**Figure 4:**
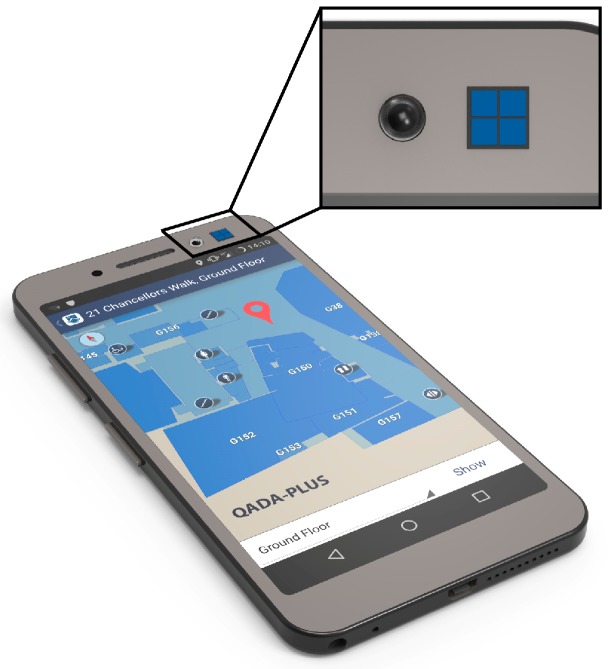
QADA-plus in a typical smartphone. The inset shows the QADA sensor located next to the standard front-facing camera.

Figure 3 demonstrates how the two sensors work together in the QADA-plus receiver. LEDs installed in the ceiling of a room are transmitting information about their position in a local or global coordinate system, as shown in Figure 1. We call this transmitted information the beacon information packet (BIP). The transmitted signals are designed to be orthogonal and thus signals from multiple luminaires can simultaneously be separated by the QADA receiver. For each luminaire, QADA detects the angle of the incoming light and decodes the transmitted data. The position of each luminaire, along with the respective AOA, is then sent to a processing unit. While QADA is estimating the AOAs and decoding the data, a camera, with the same orientation as the QADA (as seen in Figure 4) captures an image of the luminaires on the ceiling. Using the AOAs, each luminaire that was detected by QADA is located in the image. The receiver now knows the identity and location of each luminaire and so long as sufficient luminaires are visible, triangulation from the high-resolution image occurs, providing an accurate position estimate in three-dimensions.

An example of a potential configuration of the two sensors is shown in Figure 4 where a QADA sensor is located next to the front-facing camera on a typical smartphone. This figure demonstrates an important future application for the QADA-plus. These cameras typically have a sufficient FOV and high pixel counts, making them ideal for accurate AOA estimation in the second stage. Another future configuration is a stand-alone widget that can be placed on assets or drones. For the purpose of asset and robot tracking, an additional uplink would be required to send the position back to the central control or monitoring unit. This uplink could be radio-frequency, infrared or VLC [29].

### 2.3. Reference Points

Reference points associated with luminaires, first described in [26], are a novel addition to camera-based VLP and are an important optional feature for systems using QADA-plus. A reference point is a visibly distinct location that can be detected by the imaging sensor. It is used to increase the precision when identifying the positions in the image that will be used for triangulation.

Potential reference point types may include: a single LED of a different wavelength that may or may not be visible to the human eye, a small mark on the luminaire frame or nearby to the luminaire, or a well-defined position in the luminaire, such as a corner. As a default, the reference point would be the centroid of the luminaire. Also, because the information about the reference point is transmitted by the luminaire, the particular reference point type and size that is chosen for a system can be optimized for the specific application.

**Figure 5:**
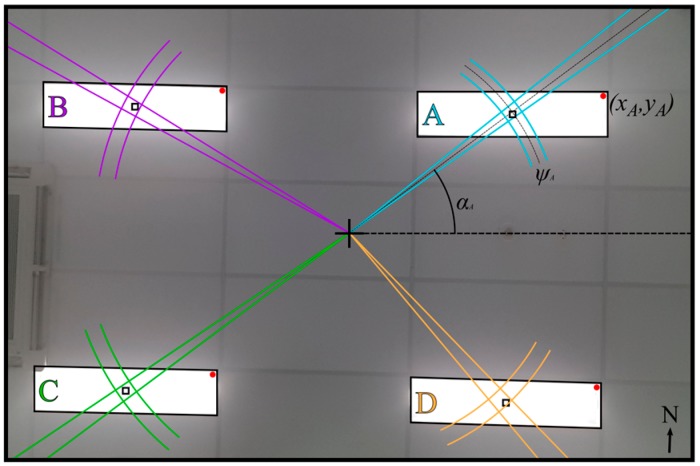
Image captured by smartphone camera showing four luminaires with a single red reference point each.

In Figure 5, an image captured from the front-facing camera of a smartphone shows four batten luminaires demonstrating reference points. Batten luminaires, as well as linear lighting, are very commonly used in large commercial and industrial buildings. In this example, the reference point is a single red LED in the corner of each luminaire, however it would be possible to have more than one reference point on each luminaire. The overlay shows the information that is provided to the second stage from the QADA sensor. The estimated AOA is used to match the luminaires to the co-ordinates which have been transmitted in the BIPs and the imaging sensor can then detect the precise positions of the reference points to use in the triangulation algorithm. The number, type and location of the reference points may be globally defined for the system or can be transmitted in the BIP.

In many buildings the luminaires are large and sparsely spaced. It can be difficult to guarantee that enough luminaires will be captured in a single image frame to support triangulation. It can also be difficult to capture the entire luminaire in the frame, thus making it challenging to determine the centroid. In the case of large luminaires, there is the option of having multiple reference points for a single luminaire, thereby reducing the number of luminaires that must be visible to allow triangulation. This means that, so long as sufficient reference points are visible, partially obscured luminaires will still contribute to the positioning accuracy as there is no requirement that all of their reference points are visible. This is very advantageous as it also relaxes the need for a very large FOV for both the imaging sensor and the QADA.

## 3. QADA Triangulation Algorithm

To estimate the position of the receiver, a triangulation algorithm is needed. QADA estimates the AOA of the light from multiple luminaires simultaneously. As long as enough luminaires are in the FOV, it is possible to triangulate from this information.

Positioning accuracy is not just affected by the accuracy of the AOA estimates but it is also sensitive to the relative positions of the luminaires and the room dimensions as this will impact the geometric dilution of precision (DOP) [30].

**Figure 6:**
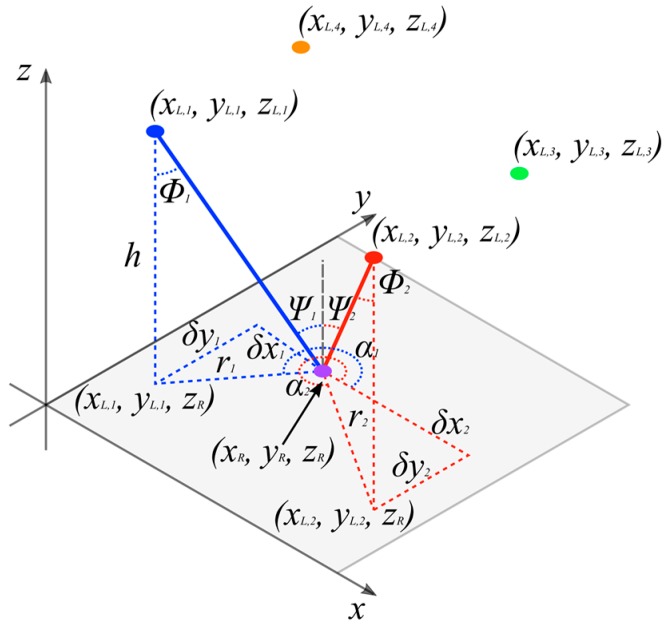
Co-ordinate system and angles used for triangulation algorithm.

The AOA is composed of two angles; the incident angle, ψ, and the polar angle, α. Figure 6 shows an indoor room scenario with four transmitting LEDs and one receiver. For this algorithm and the simulations in later sections, it is assumed that the receiver is pointing directly up towards the transmitters and thus the incident angle is equal to the emergence angle, ϕ. The transmitters are located at $\left(x_{L,n},y_{L,n},z_{L,n}\right)$, where *n* is the LED index, and the receiver is located at $\left(x_{R},y_{R},z_{R}\right)$. From the diagram, it can be seen that:

(1) $$\begin{array}{l} \delta x_{n}=x_{R}-x_{L,n}\\ \delta y_{n}=y_{R}-y_{L,n} \end{array}$$

The vertical distance from the transmitter to the receiver plane is h and the projection onto the receiver plane of the line from the *n*th transmitter to the receiver is defined as $r_{n}$. Using simple geometry, the relationship between these two variables is given by:

(2) $$\begin{array}{cc} r_{n} & =h\tan\psi_{n}\\ & =(z_{L,n}-z_{R})\tan\psi_{n} \end{array}$$

Thus, to find $x_{R}$, the relationship between $r_{n}$ and $\delta x_{n}$ can be used to give:

(3) $$\begin{array}{cc} x_{R} & =x_{L,n}+(z_{L,n}-z_{R})\tan\psi_{n}\cos\alpha_{n}\\ & =x_{L,n}+z_{L,n}\tan\psi_{n}\cos\alpha_{n}-z_{R}\tan\psi_{n}\cos\alpha_{n} \end{array}$$

The same process can be applied to find $y_{R}$. If *N* transmitters are present, an estimate of the position of the receiver can be found using linear least squares estimation:

(4) $$\hat{x}={\left(A^{T}A\right)}^{-1}A^{T}b$$

where:

(5) $$A=\left[\begin{array}{ccc} 1 & 0 & \tan\psi_{1}\cos\alpha_{1}\\ 0 & 1 & \tan\psi_{1}\sin\alpha_{1}\\ \vdots & \vdots & \vdots \\ 1 & 0 & \tan\psi_{N}\cos\alpha_{N}\\ 0 & 1 & \tan\psi_{N}\sin\alpha_{N} \end{array}\right],\;x=\left[\begin{array}{c} x_{R}\\ y_{R}\\ z_{R} \end{array}\right],\;b=\left[\begin{array}{c} x_{L,1}+z_{L,1}\tan\psi_{1}\cos\alpha_{1}\\ x_{L,1}+z_{L,1}\tan\psi_{1}\sin\alpha_{1}\\ \vdots \\ x_{L,N}+z_{L,N}\tan\psi_{N}\cos\alpha_{N}\\ x_{L,N}+z_{L,N}\tan\psi_{N}\sin\alpha_{N} \end{array}\right]$$

## 4. Analysis and Limitations of QADA

In this section, the QADA receiver is analyzed in detail and the limitations of the assumptions made are highlighted. The received optical power, $P_{R}$, is proportional to the transmitted optical power, $P_{T}$ with the relationship $P_{R}=h_{j}P_{T}$, where $h_{j}$ is the DC channel gain. In general, luminaires have diffusers that alter the radiation pattern of the LEDs. However, for this analysis the LED transmitters are assumed to have a Lambertian radiation pattern of order *m* = 1. Thus, the channel gain for the *j*th quadrant at time, t, is given by [31]:

(6) $$h_{j}(t)=\frac{\left(m+1)A_{j}(t\right)}{2\pi d^{2}(t)}\cos^{m}\left(\phi (t)\right)T_{s}\left(\psi (t)\right)g\left(\psi (t)\right)\cos\left(\psi (t)\right)$$

where d is the distance between the transmitter and the receiver, $A_{j}\left(t\right)$ is the area of quadrant *j* which the light spot overlaps, $T_{s}\left(\psi (t)\right)$ is the signal transmission of the filter and g(ψ(t)) is the concentrator gain. We consider the case where the incident and emergence angles are equal, and the QADA does not have any optical filters or lenses, thus these values are set to 1. Therefore, the photocurrent, $i_{j}(t)$, in the *j*th quadrant at time, *t*, is:

(7) $$i_{j}(t)=\frac{RP_{T}A_{j}(t)\cos^{2}\left(\psi (t)\right)}{\pi d^{2}(t)}$$

where *R* is the responsivity of the PD.

### Light Spot Centroid Estimation

**Figure 7:**
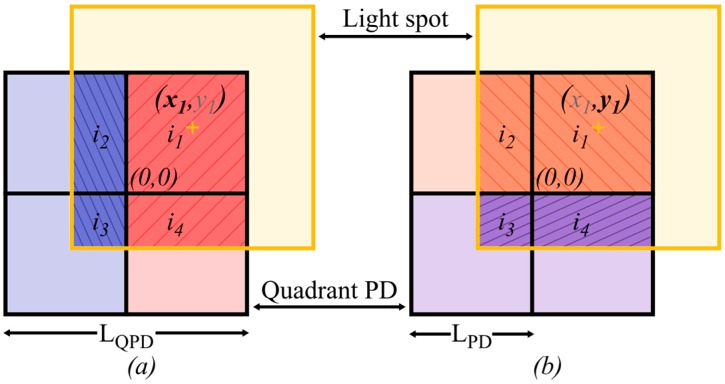
Light spot overlapping the quadrant PD. In (**a**), finding *x*_1_ uses the ratio of the red and blue segments and in (**b**), finding *y*_1_ use the ratio of the orange and purple segments.

To determine the centroid, $\left[x_{1}(t),y_{1}(t)\right]$, of the light spot, the ratio of the photocurrents from adjacent quadrant pairs is used. As the ratios depend on the relative values of the photocurrents and not the absolute values, QADA is insensitive to fluctuations in the power transmitted by the LEDs. From Equation (7), it can be seen that the photocurrent from each quadrant of the PD is proportional to the area of overlap with the light spot. This unrealistically assumes that the responsivity is uniform across the active area of the PD [32]. Thus, the ratios used to find the centroid are:

(8) $$p_{x}=\frac{i_{1}(t)+i_{4}(t)+N_{1}(t)+N_{4}(t)}{i_{2}(t)+i_{3}(t)+N_{2}(t)+N_{3}(t)}$$

(9) $$p_{y}=\frac{i_{1}(t)+i_{2}(t)+N_{1}(t)+N_{2}(t)}{i_{3}(t)+i_{4}(t)+N_{3}(t)+N_{4}(t)}$$

where $N_{j}(t)$ is the noise in the *j*th quadrant at time, *t*. This is shown in Figure 7 where the ratio of the left and right quadrant pairs is used to find $x_{1}(t)$ and the ratio of the top and bottom quadrant pairs is used to find $y_{1}(t)$. The dominant noise in the system is the shot noise due to background light. This is modelled as a white Gaussian process with zero mean and variance given by:

(10) $$\sigma_{\mathrm{shot}}^{2}=2qRp_{n}A\Delta \lambda B$$

where *q* is the charge of an electron, *p_n_* is the spectral irradiance, *A* is the area of a PD quadrant, Δ*λ* is the optical bandwidth and *B* is the electrical bandwidth.

It is important to note that the noise terms can be averaged out over many samples to reduce the errors, however manufacturing limitations will limit how well the photocurrents relate to the AOA. For example, Equations (8) and (9), assume that the individual PD quadrants are perfectly matched, which is unrealistic.

To estimate the centroid, the ratio functions are considered in the absence of noise, where they reduce to the ratio of the areas of overlap. If $L_{PD}$ is the length of a single quadrant, then Equations (8) and (9) become:

(11) $$p_{x}(t)=\frac{A_{1}(t)+A_{4}(t)}{A_{2}(t)+A_{3}(t)}=\left\{ \begin{array}{cc} \frac{L_{\mathrm{PD}}+x_{1}(t)}{L_{\mathrm{PD}}}, & -L_{\mathrm{PD}}<x_{1}(t)\leq 0\\ \frac{L_{\mathrm{PD}}}{L_{\mathrm{PD}}-x_{1}(t)}, & 0<x_{1}(t)<L_{\mathrm{PD}} \end{array}\right.$$

(12) $$p_{y}(t)=\frac{A_{1}(t)+A_{2}(t)}{A_{3}(t)+A_{4}(t)}=\left\{ \begin{array}{cc} \frac{L_{\mathrm{PD}}+y_{1}(t)}{L_{\mathrm{PD}}}, & -L_{\mathrm{PD}}<y_{1}(t)\leq 0\\ \frac{L_{\mathrm{PD}}}{L_{\mathrm{PD}}-y_{1}(t)}, & 0<y_{1}(t)<L_{\mathrm{PD}} \end{array}\right.$$

As can be seen from Equation (11), the values of $x_{1}(t)$ and $y_{1}(t)$ do not depend on each other and thus can be estimated independently. With the dependence on $L_{PD}$, the assumption again is that all the quadrants of the PD are identical. Thus, any small manufacturing imperfection will impact the accuracy of the estimation.

**Figure 8:**
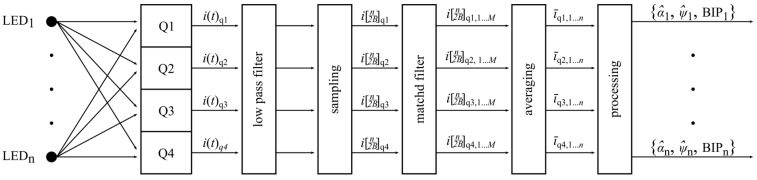
Digital signal processing path for QADA.

For each quadrant of the PD, the received signals undergo low pass filtering to limit the bandwidth to *B* hertz and are then sampled at the Nyquist rate, 2*B*. A matched filter is used to select the desired signal and reject orthogonal signals and the result is then averaged over *M* samples and substituted in Equations (8) and (9) to give an estimate at time t=(k+M)/2B. This process is shown in Figure 8. The estimate for $x_{1}(t)$ is expressed in Equation (13) and, using the same logic, a similar expression can be derived for $y_{1}(t)$.

(13) $${\hat{x}}_{1}\left[\frac{k+m}{2B}\right]=\left\{ \begin{array}{cc} L\left(p_{x}\left[\frac{k+M}{2B}\right]-1\right), & -L<x_{1}\left[\frac{k+m}{2B}\right]\leq 0\\ L-\frac{L}{p_{x}\left[\frac{k+M}{2B}\right]}, & 0<x_{1}\left[\frac{k+m}{2B}\right]<L \end{array}\right.$$

## 5. AOA Estimation for QADA

In this section, simulation results for AOA estimation are presented. It is important to note, that whilst the simulation parameters have been chosen to be realistic, the simulations do not take into account all sources of error. To be clear about the contributions of different sources of error to the limitation of QADA, we start by considering the case where the only source of error is noise. We assume the ideal case of a point source and a QADA with none of the limitations discussed in the previous section. Our previous experimental work in [20] demonstrates the limitations of assuming ideal cases in simulations. Next is an investigation on the impact of changing the size and shape of the LED transmitter. It is obvious that in the presence of multiple realistic sources of error, the performance of the receiver will be worse than the results presented below.

The parameters used in the simulations are shown in Table 1. The noise equivalent power is taken from the datasheet of a Hamamatsu quadrant PD [33] and the spectral irradiance is widely reported in the literature [31].

### 5.1. Angle of Arrival Accuracy for a Point Source

**Figure 9:**
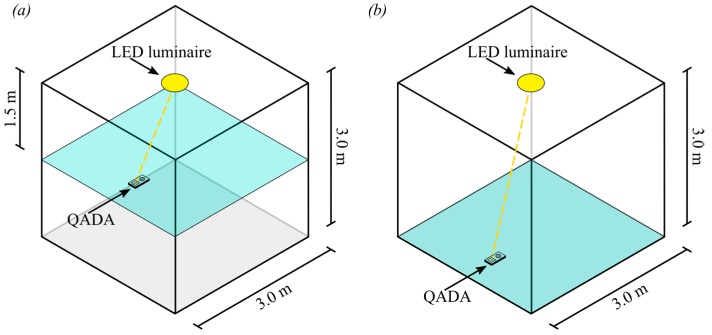
Two different room configurations used for the simulations. In (**a**) the vertical distance between the transmitter and the receiver is 1.5 m and in (**b**) the vertical distance between the transmitter and the receiver is 3.0 m.

The ability of the QADA receiver to estimate the incident and polar angles is first evaluated. Initially, the room configuration in Figure 9a is used where a single luminaire, transmitting 3 W of optical power, is located in the center of the ceiling of a room with dimensions 3.0 m × 3.0 m × 3.0 m. After low pass filtering, the transmitted signal is sampled at the Nyquist rate and averaged over 10 milliseconds, resulting in 20,000 samples used for a single estimate. The incident and polar angles are estimated for all positions in the room at a vertical distance of 1.5 m from the ceiling. We do not consider the effect of reflected light from the walls. This scenario represents an ideal case for indoor positioning with the receiver pointing directly up and the luminaires modelled as point sources.

**Figure 10:**
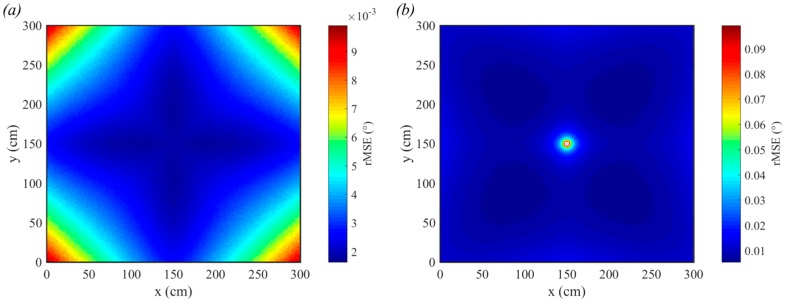
rMSE for incident angle detection (**a**) for polar angle detection (**b**). The transmitted power is 3 W, the vertical distance from the transmitter to the receiver is 1.5 m and each estimate is the average of 20,000 samples.

The results shown in Figure 10 demonstrate the general trends of the root-mean-square-error (rMSE) for incident (a) and polar (b) angles. The values of the rMSE are very small only because a large number (20,000) of samples were used. It can be seen that the incident angle estimation is least accurate in the corners of the room and the polar angle estimation is worst in the center of the room. Also, from Figure 10b, it can be seen that the polar angles close to 45 + 90*n*° (where *n* is any integer) have lower rMSE than those closer to 0 + 90*n*°. This effect is much less noticeable than the large peak in the central values.

**Figure 11:**
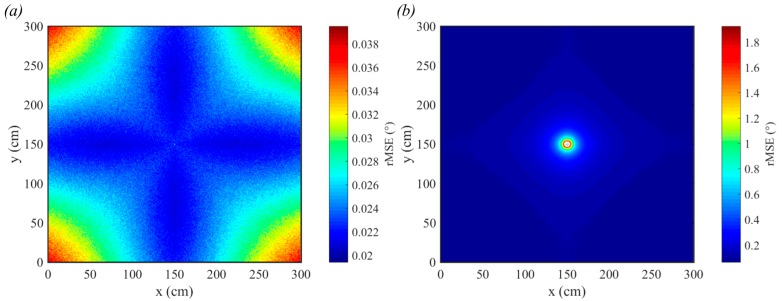
rMSE for incident angle detection (**a**) for polar angle detection (**b**). The transmitted power is 1 W, the vertical distance from the transmitter to the receiver is 3.0 m and each estimate is the average of 20,000 samples.

To demonstrate the sensitivity of QADA to reduced received optical power, the height was increased to 3.0 m and the transmitted power of the LED was reduced to 1 W. This room configuration is shown in Figure 9b. The results, in Figure 11, show that the rMSE has increased significantly for both incident (a) and polar angle (b) estimation. In particular, the polar angle estimation in the center of the room has worsened with an rMSE of almost 1.8°. Although the magnitude of the rMSE seem small, these simulations assume unrealistic ideal circumstances. The only source of error is the noise.

**Figure 12:**
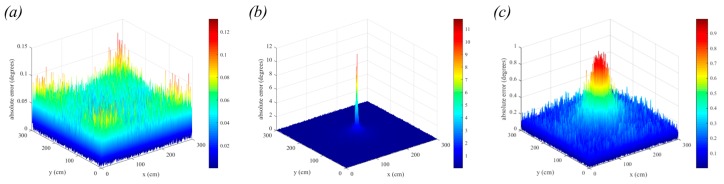
Single trial absolute error for incident angle detection (**a**) and polar angle detection (**b**). In (**c**), the large errors in the center of (**b**) have been removed to show additional detail. The transmitted power is 1 W, the vertical distance from the transmitter to the receiver is 3.0 m and each estimate is the average of 20,000 samples.

Figure 12 uses the same room configuration as used in Figure 11, however the results show the absolute error in AOA estimation. The absolute error in incident angle estimation is shown in Figure 12a and the absolute error in polar angle estimation is shown in Figure 12b. In Figure 12c we show a truncated version of Figure 12b where the errors greater than 1° have been removed to reveal additional detail. The incident angle maximum error is 0.13° and the mean error is 0.02°. For polar angle, the maximum error is 11.78° and the mean error is 0.08°. In both cases, the minimum error is extremely close to zero. The polar angle is particularly inaccurate in the center of the room because, for these positions, small estimation errors in the centroid position have the potential to cause large errors in polar angle estimation. This data demonstrates that to achieve the greatest accuracy, the results must be averaged over a large number of samples.

### 5.2. Impact of Luminaire Size and Shape on AOA Estimation

The previous simulations made the unrealistic assumption that the transmitting LED was modelled as a point source. The following results show a more realistic scenario where the transmitter is a LED luminaire that has a finite size and shape. We investigate the effect that changing the size and shape of this LED luminaire has on the ability of the QADA to estimate the AOA and how this reduces the accuracy that can be achieved. This reduction in accuracy is because the receiver has no knowledge of the luminaire geometry, thus, to be able to estimate the AOA it assumes the luminaire is a point source. Three different common luminaire shapes are compared: square, circular and rectangular. For the square shape, the effect of increasing the size of the luminaire is also shown. Each luminaire is modelled as an array of 2500 equally spaced Lambertian transmitters. We do not consider the impact of diffusers, though, in practice, diffusers are carefully designed to meet lighting requirements with minimum power usage [34]. The parameters used are the same as those in Table 1 and the room configuration is that shown in Figure 9a.

#### 5.2.1. Varying Luminaire Size

**Figure 13:**
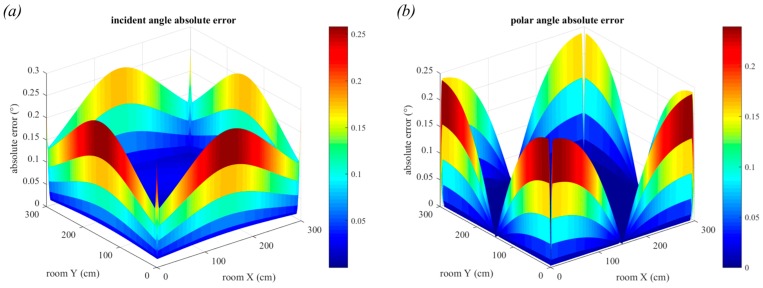
Absolute error in incident angle estimation (**a**), and in polar angle estimation (**b**), for a square luminaire with side length 10 cm.

Figure 13 shows the absolute errors for incident (a) and polar angle (b) estimation when a small square luminaire with side length 10 cm is used. For the incident angle, the errors in the outer edges of the room dominate, whilst for the polar angle the errors are greatest in the corners. In both cases, the maximum estimation errors are close to 0.25° (0.26° for incident angle and 0.24° for polar angle). The large errors in the extremities of the room are because the entire luminaire is not in the FOV of the QADA in these locations. This demonstrates one of the limitations of QADA.

**Figure 14:**
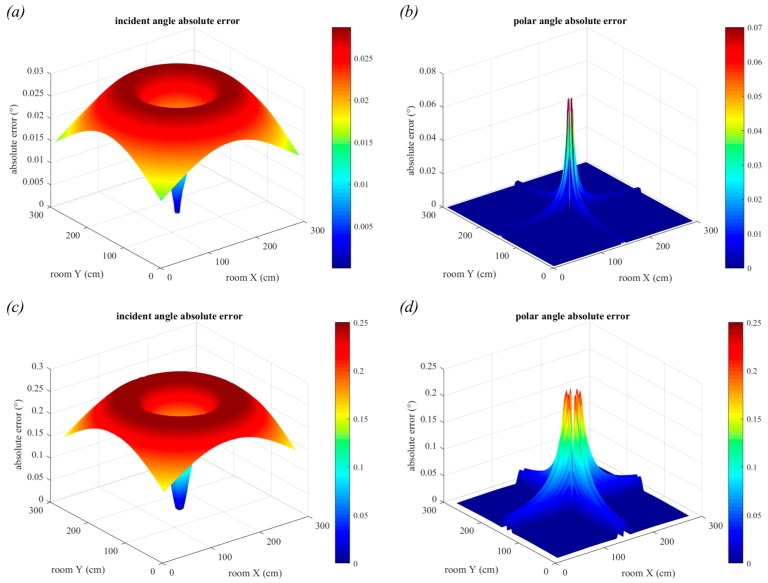
Absolute error in incident angle estimation and in polar angle estimation for a square luminaire with side length 10 cm, (**a**,**b**), and for a square luminaire with side length 30 cm, (**c**) and (**d**).

In Figure 14a,b the outer edges of the room are omitted so that the smaller estimation errors in the central part of the room are clearer. In this region, the incident angle estimation is most accurate directly below the luminaire, whilst the polar angle is least accurate. The polar angle estimation experiences decreased estimation accuracy in the regions close to 0 + 90n°, whilst for all other parts of the room, the error is close to 0°. The mean incident angle error is 0.026°, whilst the mean polar angle error is close to 0°.

In Figure 14c,d the size of the square luminaire has been increased to 30 cm. Whilst the overall trend remains the same, the maximum estimation errors have increased to 0.86° for the incident angle and 0.68° for the polar angle. It can be seen that the maximum incident angle error is 0.26°, a tenfold increase from the smaller 10 cm luminaire, and the maximum polar angle error is 0.22°, also a very large increase. The mean errors are 0.22° for incident angle and 0.008° for polar angle. Again, the results show the effect of one source of error only. Thus, whilst the errors may seem small, the cumulative effect of all the errors must be considered. These results are primarily to highlight the trends and to demonstrate the degradation in performance as parameters are varied.

#### 5.2.2. Varying Luminaire Shape

**Figure 15:**
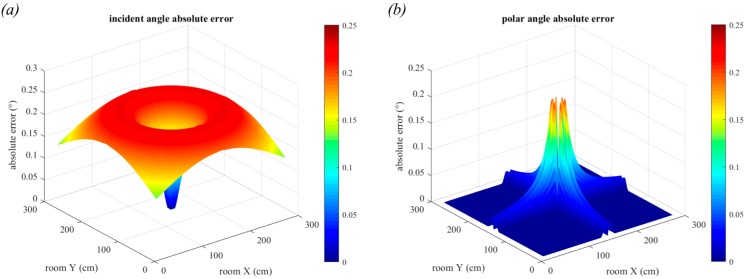
(**a**) Absolute error in incident angle estimation, and (**b**) in polar angle estimation for a circular luminaire with diameter 30 cm.

In this section, the figures show only the portions of the room where the receiver can see the entire luminaire in the FOV. The results for a circular luminaire with diameter 30 cm are shown in Figure 15. Both this figure and the corresponding subfigures (c) and (d) from Figure 14 are on the same scale, allowing for direct comparison. Interestingly, the results are very similar to those of the square luminaire with side length 30 cm. However, the circular luminaire does perform marginally better than the square luminaire. The maximum errors for incident angle estimation are 0.23° and for polar angle estimation are 0.21°. The mean errors in this region are 0.19° for incident angle and 0.006° for polar angle. The reason the circular luminaire performs better than the square luminaire is because the circular one has a smaller area than the square. Later, we will show the effect this small improvement has on positioning error.

**Figure 16:**
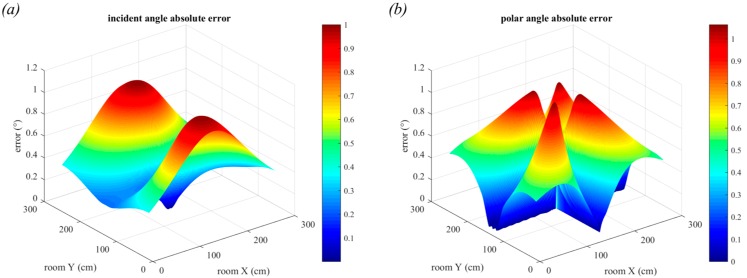
(**a**) Absolute error in incident angle estimation, and (**b**) in polar angle estimation for a rectangular light with dimensions 30 cm × 60 cm.

The dimensions that are used for the rectangular luminaire are 30 cm × 60 cm, with the long side aligned with the *x*-axis. The results for this luminaire are shown in Figure 16. It can be seen that they are significantly worse than those of the square and circular, with the maximum error in incident angle of 1° and for polar angle, the maximum error was 1.06°. The results are worse along the *x*-axis is because it is aligned with the longer side of the rectangle. The performance degradation seen with the rectangular luminaire is because it has fewer planes of symmetry than the square and the circular ones.

## 6. Triangulation Simulations Using QADA

**Figure 17:**
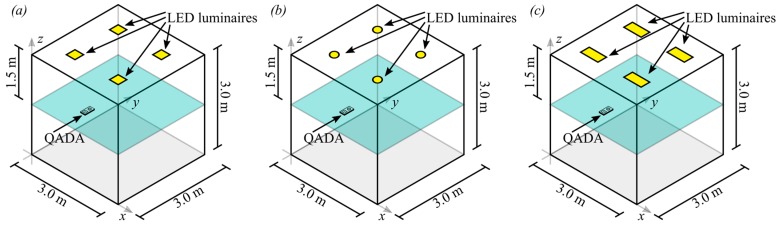
Room configurations for triangulation simulations. In (**a**), square luminaires with side length 30 cm are used, in (**b**), circular luminaires with diameter 30 cm are used and in (**c**), rectangular luminaires with dimensions 30 cm × 60 cm were used. In all cases, the centers of the luminaires are 75 cm from the edges of the room.

The ultimate goal of QADA is not AOA estimation, but positioning, and thus this section details the effect that the errors in AOA estimation have on positioning accuracy. Positioning error is defined as the Euclidean distance from the estimated position to the true position. In the following simulations, the parameters used are in Table 1, the triangulation algorithm is from Section 3 and the room configuration is shown in Figure 17. In all three cases, the centroids of the luminaires are located at {(75, 75, 300), (75, 225, 300), (225, 75, 300), (225, 225, 300)} cm. These positions are chosen so that the luminaires are evenly distributed in the room. This is not entirely realistic as luminaires are often not evenly spaced. Again, we consider a best-case scenario and different configurations and receiver orientation would give worse results.

Measurements are taken for all positions on a plane that is 1.5 m below the luminaires. To ensure the entirety of each luminaire is in the FOV of the QADA in all positions, the aperture height is changed to 1.25 mm. We have previously shown in [25] that increasing the FOV of QADA will result in worse performance in the presence of noise.

### 6.1. Triangulation in the Absence of Noise

**Figure 18:**
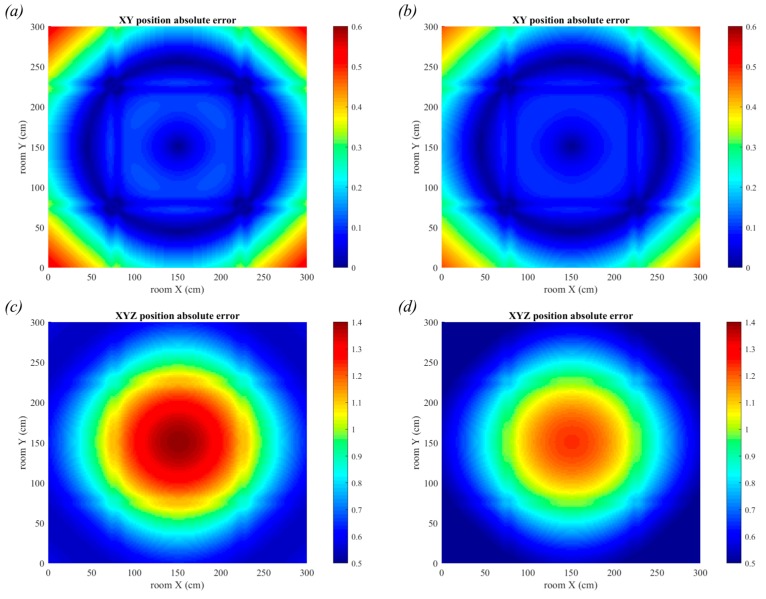
Positioning error for square luminaires with side length 30 cm, (**a**,**c**), and circular luminaires with diameter 30 cm, (**b**,**d**). Errors in two-dimensions are shown in (**a**,**b**) and errors in three-dimensions are shown in (**c**,**d**).

Initially, the simple case with no noise is considered, thus the only source of positioning error is the size and shape of the luminaire. Figure 18 shows the positioning errors for the scenarios in Figure 17a,b with the square and circular luminaires, respectively. There are many positioning applications that do not require three-dimensional positioning and thus in Figure 18a,b the errors in only the *x-y* plane are considered. The errors are much smaller if only two-dimensions are considered, with the maximum error of 0.53 cm for the square luminaires and 0.47 cm for the circular luminaires. In contrast, the maximum error in three-dimensions was 1.3 cm for the square luminaires and 1.24 cm for the circular luminaires. When only two-dimensions are considered, the largest errors are in the corners of the room, however in three-dimensions the largest error is in the center of the room. The corners have very similar errors in both cases, but the errors in the center of the room have greatly increased in three-dimensional positioning. This is caused by the contribution from the error in the estimation of the *z* co-ordinate which is large in the center of the room and small in the corners. It might appear from these results that QADA alone is capable of providing accurate positioning, however it should be noted that these results are only showing the impact of a single source of error.

**Figure 19:**
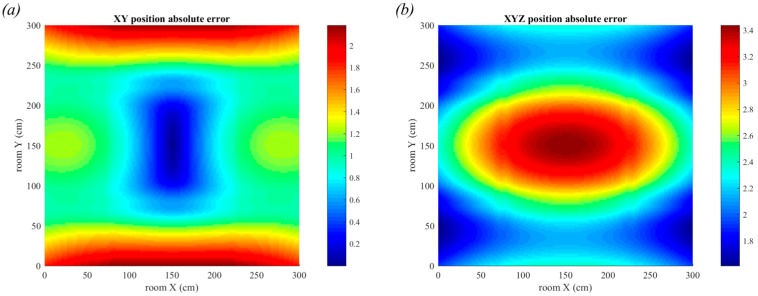
Positioning error for rectangular luminaires with dimensions 30 × 60 cm. Errors in two-dimensions are shown in (**a**) and errors in three-dimensions are shown in (**b**).

For comparison, the error when using rectangular luminaires is shown in Figure 19, using the configuration shown in Figure 17c.

The large errors in AOA estimation have led to large errors in position estimation, as expected. The maximum error in two-dimensions is 2.18 cm and in three-dimensions is 3.43 cm. Unlike noise, these errors cannot be reduced by averaging over many samples. This is a particularly poor performance in two-dimensions, almost four times worse than using square or circular luminaires, and it suggests that rectangular luminaires are not ideal for use with the QADA receiver.

### 6.2. Triangulation in the Presence of Noise

**Figure 20:**
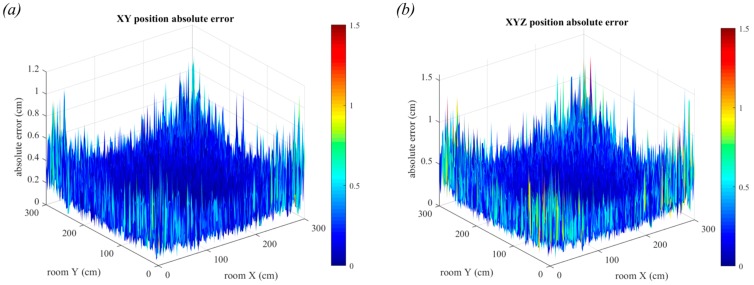
Positioning error in the presence of noise with point source transmitters. Errors in two-dimensions are shown in (**a**) and errors in three-dimensions are shown in (**b**). Each estimate is the average of 20,000 samples.

The effect of noise on the positioning error is presented in this section. Initially, the transmitters are assumed to be point sources and all other room dimensions are the same as those in Figure 17. In reality, these room dimensions are very small compared to most indoor scenarios. The largest errors are in the edges of the room, as seen in Figure 20. In these positions, the light from three of the four luminaires will have travelled long distances, leading to worse AOA estimation. The maximum errors for two and three-dimensions are quite similar, however the mean in two-dimensions is lower at 0.14 cm compared to 0.21 cm for three-dimensions. It can also be seen that there is more variability in the errors for three-dimensions, particularly in the edges of the room.

**Figure 21:**
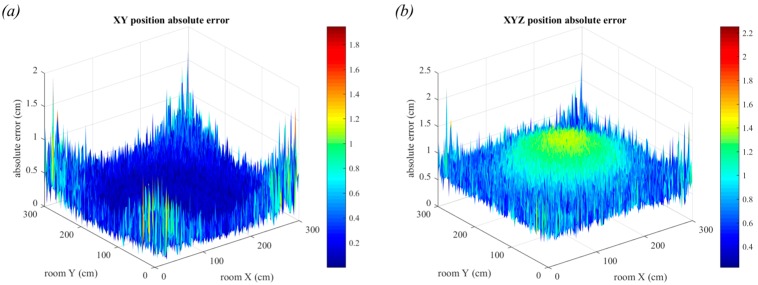
Positioning error in the presence of noise with square luminaires and a vertical distance of 1.5 m. Errors in two-dimensions are shown in (**a**) and errors in three-dimensions are shown in (**b**). Each estimate is the average of 20,000 samples.

In Figure 21, the noise parameters remain the same, but the luminaires are no longer point sources. The room configuration used is shown in Figure 17a, with square luminaires of side length 30 cm.

In Figure 21a, the positioning error in two-dimensions is shown. The error has increased to a maximum of 1.9 cm and from the shape of the plot, it can be seen that the large central region is very flat, much like in Figure 18a. This shows the strong effect of having the large luminaires for this room configuration. In Figure 21b, this trend is more obvious with the large central area of increased error when compared to Figure 20b. This trend was previously seen in Figure 18c.

**Figure 22:**
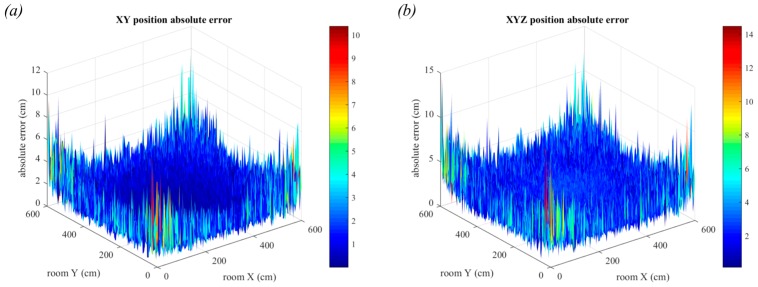
Positioning error in the presence of noise with square luminaires, room dimensions 6 m × 6 m and a vertical distance of 3 m. Errors in two-dimensions are shown in (**a**) and errors in three-dimensions are shown in (**b**). Each estimate is the average of 20,000 samples.

In Figure 22, the receiver position is changed to be 3 m below the ceiling and the room dimensions have changed to 6.0 m × 6.0 m. It can now be seen that the positioning error due to luminaire size is less evident and that the noise appears to be the dominant source of error. This is clearest when comparing Figure 21b with Figure 22b where the previously seen central trend is no longer visible. The reason for this is because when the room dimensions increase but the luminaire size remains the same, the ratio of the two values will have decreased and thus the overall impact of luminaire size is lessened. However, the increased distance the light has travelled results in a much lower received optical power, resulting in increased positioning error. The maximum error in two-dimensions is now 10.38 cm and the maximum error in three-dimensions has increased to 14.47 cm.

**Figure 23:**
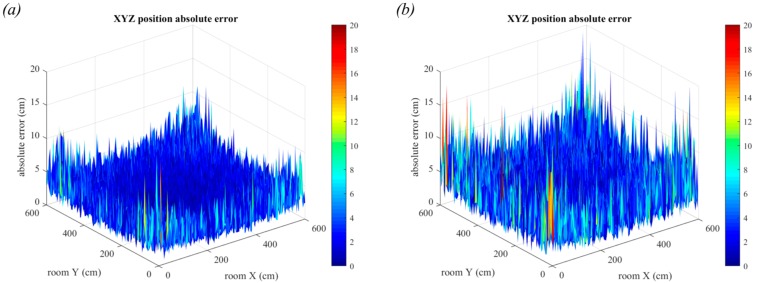
3-dimensional positioning error in the presence of noise with square luminaires and room dimensions 6 m × 6 m. In (**a**) the vertical distance is 4 m and in (**b**) the vertical distance is 5 m. Each estimate is the average of 20,000 samples.

When the vertical distance between transmitter and receiver is increased, the performance degrades further due to the lower optical power received. In Figure 23, we show the 3-dimensional positioning errors for two additional vertical distances in the 6 m × 6 m room. In Figure 23a, the vertical distance is 4 m and in Figure 23b, the vertical distance is 5 m. These are reasonable distances to expect in large buildings with high ceilings. The maximum errors have now increased to 16.37 cm and 20.51 cm, respectively. Whilst these large errors are primarily in the corners of the room, these results demonstrate the progressive decline in QADA performance as the simulation parameters approach reality.

### 6.3. Triangulation with Fewer Samples

**Figure 24:**
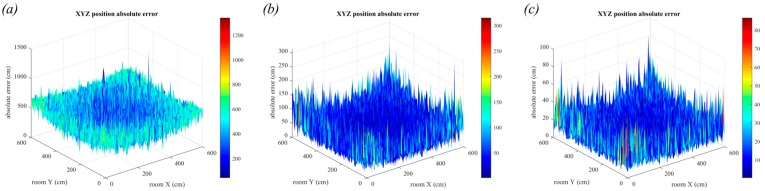
3-dimensional positioning error in the presence of noise with square luminaires and room dimensions 6 m × 6 m × 5 m. Position estimates are calculated using (**a**) a single sample, (**b**) 100 samples and (**c**) 1000 samples. Note that these figures are on different scales.

In the previous simulations, with the assumption of a perfectly calibrated and aligned receiver, it is clear that the noise is the dominant source of error, and the system is relying heavily on averaging over a large number of samples to achieve acceptable results. In many situations it may not be possible to average over many samples, such as power constrained systems that cannot sample at very high frequencies or when position update frequency requirements are high, limiting the time available for sampling. In Figure 24 we show the results when fewer samples are used to calculate the estimate. In Figure 24a a single sample was used, and it can be seen that the errors are very large with a mean positioning error of 4.4 m. In Figure 24b, the estimate uses an average of 100 samples resulting in a mean positioning error of 45 cm and in Figure 24c, the estimate uses an average of 1000, resulting in a mean positioning error of 14.4 cm. These values can be compared to Figure 23b where 20,000 samples were used, giving a mean positioning error of 3.25 cm. An advantage of adopting a two-stage system is that the requirement to use a large number of samples is relaxed because the system does not rely on the QADA receiver for accuracy.

Again, it is important to remember these results, along with all the results in this paper, are simulation only and unrealistically assume near ideal conditions. It was demonstrated that as the simulation model increased in complexity, with the addition of more potential sources of error, that the results degraded. It is reasonable to assume that if more potential sources of error were to be added to the simulation model, that the performance can only get worse. In our experimental work with the QADA receiver [20], we showed that our simulations underestimated the rMSE in incident angle estimation by up to a factor of two over very short distances.

## 7. Discussion and Conclusions

We have introduced the QADA-plus receiver, a two-stage receiver that has been optimized for VLP. This receiver consists of two sensors–a PD-based AOA detector and an imaging sensor. QADA-plus has the advantage of being compatible with modern consumer electronics due to its compact, planar structure. The unique feature of this receiver is that it combines the data from two different optical sensors, thereby taking advantage of the relative strengths of each sensor.

### 7.1. Accuracy of QADA as a Stand-Alone Sensor

The accuracy of PD based VLP systems is fundamentally limited by the small number of PDs and the resulting requirement for very accurate matching and alignment of components and very accurate measurement of the signal received by each PD. The QADA receiver has important advantages over some other PD receivers, including a planar structure, the use of a single aperture, and the use of a quadrant PD in which the properties of the PDs are closely matched. However, despite these advantages, it cannot overcome the fundamental limitation of PD based systems and we have previously shown that even in a careful experiment with transmission over a short distance there is significant discrepancy between measurement and simulation [20].

Two different factors limit the performance of a QADA: noise and systematic errors. Our detailed results demonstrate the potential accuracy under ideal conditions of the QADA receiver when used as a stand-alone sensor. We have presented extensive simulations using realistic parameter values showing that, by averaging over a number of measurements, the errors caused by noise alone can be made very small. An advantage of PD based systems is that they can be designed with relatively high bandwidth so very many samples can be taken within a short period of time. A disadvantage of averaging over a large number of samples is the computational cost and energy consumption.

We have provided results for one source of systematic error–the fact that typical luminaires are not point sources. Results are given for round, square and rectangular luminaires. Increasing the size of the luminaire is shown to increase the positioning error throughout the room, but it is particularly large near the walls where the entire luminaire is not within the FOV of the receiver. This highlights one limitation of PD based systems–their sensitivity to partial blocking of transmission from a luminaire. This will often occur in practice when a person or piece of equipment or furniture obscures part of the luminaire. The precise design of the diffuser of a luminaire will also significantly affect the radiation pattern. Luminaire manufacturers carefully design the diffuser to meet the illumination requirements with minimum power consumption.

In the simulated scenarios, the maximum error in three-dimensional positioning in the presence of noise was 20.51 cm using square luminaires. These results considered only the effect of luminaire geometry and noise, thus in practice, QADA is unlikely to achieve this level of accuracy.

### 7.2. Other Sources of QADA Error

There are also many other potential sources of error. These include limitations in the manufacturing of the QADA, changes to the QADA with time and use such as PD ageing [35] and responsivity variation with temperature, the properties of real luminaires, the orientation of the receiver and the locations of the luminaires.

The AOA estimation in the QADA is based on the ratio of the photocurrents from the four quadrants of the PD and assumes that each photocurrent is directly proportional to the illuminated area of that quadrant. This depends on perfect alignment of the aperture and the PD, perfect uniformity of response across the entire surface of all four PD quadrants, and perfect matching of the output circuits for each quadrant. While some of these effects may be mitigated by a calibration step at the production stage, they may be subject to change with time and variations in temperature which calibration cannot correct. The balance may also be affected by dirt on the surface of the aperture and small changes to the surface such as scratches. In the simulations we used an ideal scenario, in which the luminaires were evenly spaced, and the receiver was pointing upwards. It is well known that in less ideal situations DOP significantly reduces the accuracy that can be achieved. The QADA, like other positioning systems, shares this limitation. The layout of luminaires in buildings is determined by lighting requirements, aesthetics and infrastructure design and in many cases will not be ideal. Also, any tilting of the receiver will affect which luminaires are within its FOV.

### 7.3. Reference Points

We have also introduced the concept of reference points; visibly distinct features associated with luminaires for use with QADA-plus. While the importance of reference points may not be immediately obvious–they offer multiple important practical advantages. Their measured position does not depend on the size and radiation pattern of a luminaire, therefore removing the problem of partial obstruction of luminaires. There can be multiple reference points per luminaire–so fewer luminaires need to be within the FOV of the receiver. The size and shape of reference points can be designed to optimize their identification by the camera part of the QADA-plus. For example, it may be advantageous to choose shapes for which there are well known image processing algorithms, such as circles. The use of reference points depends on the ability of the QADA-plus to both receive a significant amount of data from each luminaire and to process an image to identify a small reference point. Reference points allow for flexible implementations and increased accuracy, especially in challenging environments such as buildings with large sparsely located luminaires.

### 7.4. Image Sensor Stage of QADA-Plus

The detailed requirements for the camera used for the image processing stage of the QADA are an area for further study, but it is clear that these will be less stringent than for cameras currently used for VLP and also for the fiducial marker systems used in robotics. There is no requirement for a rolling shutter in the QADA-plus—although a rolling shutter camera can be used. The image resolution required may be less than for fiducial marker systems because only the position, not the detailed structure of the reference points must be determined. While the QADA-plus is also subject to DOP, the triangulation is based on much more accurate AOA estimates, so the position estimates are also much more accurate. Thus, we can confidently assume that the experimental results found in other studies will be matched by the QADA-plus.

### 7.5. QADA-Plus

We have shown that in practice the QADA alone will provide limited positioning accuracy but despite this in situations where accuracy is not paramount, it may be adequate. It does have the benefit of being low power, very compact and offers reasonable accuracy. However, when increased accuracy and flexibility in implementation is required, the option is available to move to the two-stage QADA-plus that uses both a QADA and an imaging sensor.

In the two-stage QADA-plus, the accuracy is now related to the camera quality. A common use case is indoor pedestrian navigation, where the front facing camera on a smartphone makes an ideal second stage imaging sensor. Such cameras are high resolution and have been shown to offer good positioning accuracy [36]. In this case the positioning update rate is tied to the maximum frame rate of the camera which is typically around 30 fps. This is more than adequate for indoor pedestrian navigation. However, it may not be suitable for faster moving objects such as drones working in future factories. For more specialized applications, like drones, a better imaging sensor may be warranted to offer improved positioning accuracy and faster updates. Importantly though, with QADA-plus, the need for high frame rates is tied only to the need for fast position updates and not to the communications aspects of the system. Thus, for standard consumer needs, it is possible to use common off-the-shelf components to make a relatively cheap VLP receiver.

The QADA-plus is a promising new receiver design which by combining a PD based sensor and a camera-based sensor builds on the advantages of each. It has the potential to provide an indoor position system which like the outdoor GPS is accurate, ubiquitous and does not require access to other sources of information or prior knowledge about its location.

## 8. Patents

J. Armstrong, A. Neild, S. Cincotta, Visible light positioning receiver arrangement and two stage positioning method, 2018902351, 2018.

## Figures and Tables

**Table 1:** Simulation Parameters.

Parameter	Value
Quadrant PD size	5 mm × 5 mm
Aperture height	2.5 mm
QADA FOV	90°
Responsivity	0.25 A/W
Electrical bandwidth	1 MHz
Optical bandwidth	300 nm
Noise Equivalent Power	1.9 × 10^−14^ $W/\sqrt{\mathrm{Hz}}$
Spectral irradiance	6.2 × 10^−6^ W/(nm·cm)^2^
